# Recent Advances in Self-Assembly and Application of *Para*-Aramids

**DOI:** 10.3390/molecules27144413

**Published:** 2022-07-09

**Authors:** Chunjie Xie, Shixuan Yang, Ran He, Jianning Liu, Yuexi Chen, Yongyi Guo, Zhaoxia Guo, Teng Qiu, Xinlin Tuo

**Affiliations:** 1 Key Laboratory of Advanced Materials (Ministry of Education), Department of Chemical Engineering, Tsinghua University, Beijing 100084, China; xiechunjie@mail.tsinghua.edu.cn (C.X.); yangsx20@mails.tsinghua.edu.cn (S.Y.); her20@mails.tsinghua.edu.cn (R.H.); gasperliu97@163.com (J.L.); chenyuex21@mails.tsinghua.edu.cn (Y.C.); guo-yy21@mails.tsinghua.edu.cn (Y.G.); guozx@tsinghua.edu.cn (Z.G.); 2 Key Laboratory of Carbon Fiber and Functional Polymers (Ministry of Education), Beijing University of Chemical Technology, Beijing 100029, China; qiuteng@mail.buct.edu.cn

**Keywords:** poly (*p*-phenylene terephthalamide), liquid crystal polymer, self-assembly, nanofiber, all-aramid material

## Abstract

Poly(*p*-phenylene terephthalamide) (PPTA) is one kind of lyotropic liquid crystal polymer. Kevlar fibers performed from PPTA are widely used in many fields due to their superior mechanical properties resulting from their highly oriented macromolecular structure. However, the “infusible and insoluble” characteristic of PPTA gives rise to its poor processability, which limits its scope of application. The strong interactions and orientation characteristic of aromatic amide segments make PPTA attractive in the field of self-assembly. Chemical derivation has proved an effective way to modify the molecular structure of PPTA to improve its solubility and amphiphilicity, which resulted in different liquid crystal behaviors or supramolecular aggregates, but the modification of PPTA is usually complex and difficult. Alternatively, higher-order all-PPTA structures have also been realized through the controllable hierarchical self-assembly of PPTA from the polymerization process to the formation of macroscopic products. This review briefly summarizes the self-assembly methods of PPTA-based materials in recent years, and focuses on the polymerization-induced PPTA nanofibers which can be further fabricated into different macroscopic architectures when other self-assembly methods are combined. This monomer-started hierarchical self-assembly strategy evokes the feasible processing of PPTA, and enriches the diversity of product, which is expected to be expanded to other liquid crystal polymers.

## 1. Introduction

Poly(*para*-phenylene terephthalamide) (PPTA) is a kind of liquid crystal polymer prepared generally by polycondensation of *p*-phenylene diamine and terephthaloyl chloride at a low temperature. The products made from PPTA are also named *para*-aromatic amides, or in the abbreviation form *para*-aramids. In the 1970s, DuPont first dissolved PPTA in concentrated sulfuric acid when preparing a liquid crystal solution, and then the liquid crystal solution was fabricated into *para*-aramid fibers by a specially designed dry-jet wet-spinning process. *Para*-aramid fibers are widely used in aerospace, defense and military industries, and many important civil fields owing to its characteristics of high strength, high modulus, high temperature resistance, corrosion resistance and high dimensional stability [1,2,3]. Nevertheless, the “infusible and insoluble” characteristic of PPTA, which is attributed to its molecular rigidity, strong intermolecular hydrogen bonding and conjugation, makes it difficult to be processed via traditional methods. As a result, the fiber is the only product form that has been successfully industrialized on a large scale at present. Furthermore, the performance of *para*-aramid-fiber-enhanced composites is also challenged by the weak interfaces between *para*-aramid fibers and the matrix [4,5]. The development of novel PPTA adaptive processing strategies is practical and urgent.

Self-assembly refers to the behavior of basic structural units, including molecules and nanomaterials, that spontaneously organize or aggregate into higher-order structures under non-covalent bonding interactions [6,7,8,9,10]. The rigid planar conjugated structure and strong intermolecular interactions endow PPTA with strong tendencies in terms of orientation and self-assembly; however, the study is much retarded by the poor solubility of PPTA. Recently, interest in the self-assembly of PPTA has been relit by aramid nanofibers. No matter whether the aramid nanofibers are made with the “top down” or “bottom up” method, the essential structure consists of ordered aggregation of PPTA at the nano-scale. Therefore, retroactive research on PPTA self-assembly strategies over the years should be important and suggestive for the further design of PPTA or more high-performance polymers.

## 2. Self-Assembly of PPTA

**Figure 1:**
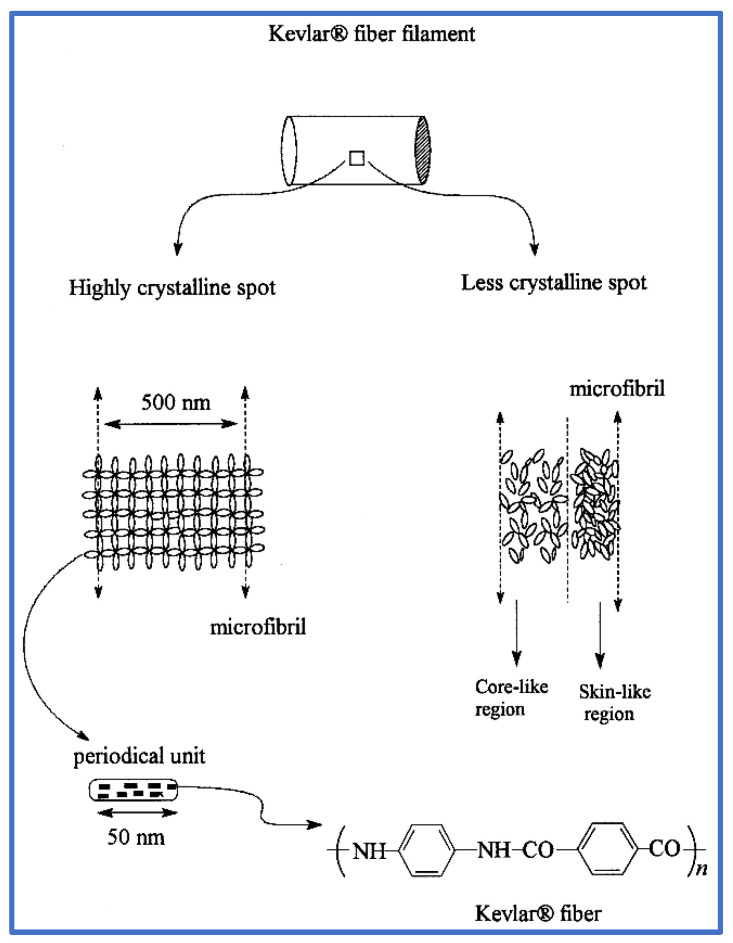
The microfibril and pleated appearance of periodical structure arrangements of aramid fiber [12]. Copyright 1999 Elsevier B.V (Amsterdam, The Netherlands).

The rigid rod-like structure lacking the flexibility of soft segments is facilitated by the planar conjugation and ordered hydrogen bonding of PPTA molecules, but also results in poor solubility, and PPTA can only be dissolved in concentrated sulfuric acid. The famous Kevlar^®^ fibers are fabricated through dry-jet wet-spinning of a PPTA/concentrated sulfuric acid solution, followed by a washing, hot stretching and winding process. Various techniques are applied to analyze the structure of aramid fibers, such as diffraction, electron microscopy, atomic force microscopy and scanning tunneling microscopy. It has been more and well more recognized that the extraordinary mechanical properties of aramid fibers (which have high crystallinity) are derived from the highly ordered microstructure formed through the self-assembly of PPTA during the fabricated process. The angle between the amide and carbonyl end groups is 160°, as calculated from XRD data, and the characteristic distance of adjacent PPTA chains is 3 Å, suggesting it is the strong hydrogen bonding which links the adjacent chains to the lattice planes [11]. In addition, based on the observed result, a skin-core conceptual model of aramid fiber is well accepted. A high degree of 3D ordered crystallinity exists at the core region, and the strong 1D macromolecular orientation dominates in the skin region, which is generally non-crystalline (Figure 1). Hence, the outstanding mechanical properties of aramid fibers originate from the radially anisotropic, hierarchically ordered microstructures [12].

### 2.1. Self-Assembly of Modified PPTA

Although the strong intermolecular interactions among polymer chains and the highly ordered hierarchical microstructures suggest PPTA as favorable building blocks for self-assembly, the precise control of PPTA remains hard to realize even in the process of concentrated H_2_SO_4_ solution-based spinning. In order to achieve a more adaptive self-assembly process, chemical modification of the PPTA chain structure is regarded as one effective method.

Modification of the chemical structure of PPTA would be carried out on the side chains, such as the introduction of side groups [13,14,15,16]; graft chains [17,18,19]; and the backbone, such as the introduction of unsymmetric structures [20,21] or ether linkages [22]. Through the sacrifice in the structural regularity, the free volume or the chain flexibility is therefore increased, so that the solubility in common solvents is also improved by these modifications. Moreover, these modified aramids could be fabricated as films with excellent mechanical strength and thermal stability. For example, Liu et al. [14] prepared 4,4’-(terephthaloylbis (azanediyl))-*bis-*(2-hydroxybenzoic acid) and applied it as the co-monomer to further synthesize an aramid containing polar hydroxyl side groups. The obtained product was fabricated as a film with the tensile strength of 95 MPa via the method of solution spinning and casting. Hsiao et al. [21] reported a series of asymmetrical diaryl-ether-modified aramids, made by the phosphorylation polyamidation of various dicarboxylic acids with 5-(4-aminophenoxy)-1-naphthylamine. Those modified aramids were amorphous and readily soluble in many organic solvents. The flexible and tough films with tensile strength of 90–128 MPa and elongations at brea of 9–64% were further prepared via solution casting. However, these studies focused more on the solubility and processibility of modified aramids rather than the molecular orientation or self-assembly behavior.

**Figure 2:**
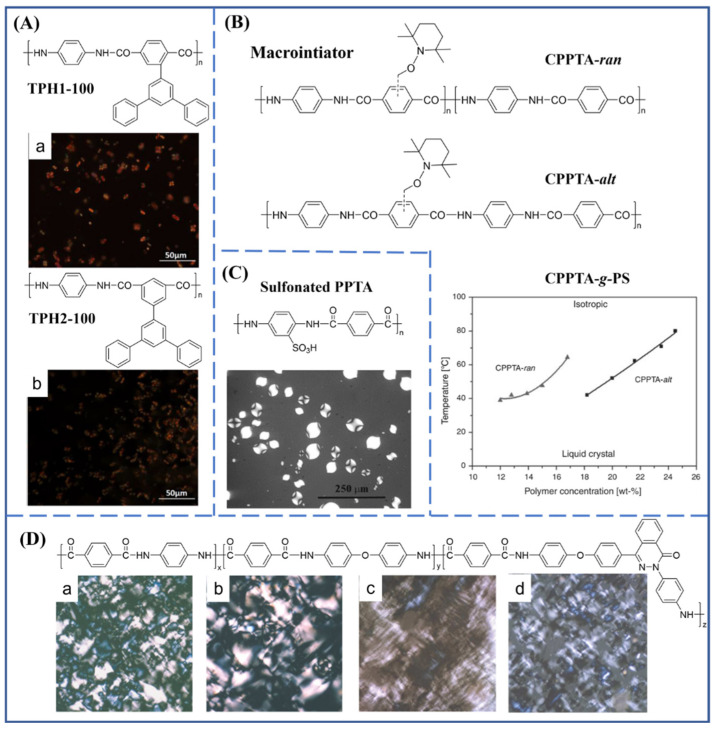
The chemical structures of modified aramids containing the side groups or graft chains and the effect of side groups or graft chains on the self-assembly behaviors of modified aramids. (**A**) Aramid containing an *m*-triphenyl pendant group; (**a**) TPH1-100 (**b**) TPH2-100. [23]. Copyright 2017 Elsevier B.V. (**B**) The TEMPO-macroinitiator for aramid with grafted polystyrene [24]. Copyright 2014 CSIRO Publishing. (**C**) Sulfonated aramid [27]. Copyright 2004 Royal Society of Chemistry (London, UK). (**D**) Aramid containing a phthalazinone moiety and ether linkages; (**a**–**d**) photomicrographs of copolyamides solution with various compositions [29]. Copyright 2005 Elsevier B.V.

The presence of side groups, bulky pendant groups or graft chains increases the spacing distance of PPTA molecular chains, and further affects the spatial orientation of the molecules, which is revealed by the evolution of liquid crystal structures. By the synthesis of two kinds of comonomers of *o*-(*m*-triphenyl)-terephthaloyl chloride and *m*-(*m*-triphenyl)-isophthaloyl chloride with bulky pendant groups, and their copolymerization with *p*-phenylenediamine (PPD) and terephthaloyl chloride (TPC), Zhou et al. [23] revealed that the lyotropic liquid crystal behaviors were influenced by the structures, molecular weights and concentrations of these modified aramids, as evidenced by polarized optical microscopy (POM) results. The representative images are shown in Figure 2A. Du et al. [24] prepared a graft copolymer of PPTA-*g*-polystyrene obtained from PPTA-based macroinitiators by nitroxide-mediated radical polymerization. The lyotropic behavior of the graft copolymer was discovered in concentrated sulfuric acid, also by POM (Figure 2B). Yun and Viale et al. [25,26,27] successively synthesized sulfonated PPTA and observed the nematic liquid crystal phase in water at very low polymer concentrations. In addition, the sulfonated PPTA with thermoreversible gelation could dissolve in a dilute aqueous solution at 80 °C and turned into a gel at room temperature. The POM result of the 1 wt% sulfonated-PPTA solution in water is shown in Figure 2C. Huang et al. [28] reported the preparation of organo-soluble aramids based on a diamine containing pyridine and carbazole groups via the Chichibabin reaction and subsequent reduction. The obtained aramid exhibited electrochromic characteristics due to the effects of solvent response and protonation. Liang et al. [29] introduced a phthalazinone moiety and ether linkages into the main chain of the aramid. The lyotropic liquid crystal behavior of the obtained modified aramid in *N*-methylpyrrolidone (NMP) was characterized by POM. The presence of a twisted non-coplanar phthtalazinone moiety and ether linkages in the modified aramid improved its solubility and helped to form a lyotropic phase in a polar organic solvent because of the increase in intermolecular freedom (Figure 2D). Seyler and Kilbinger reported the synthesis of a poly(*p*-benzamide) oligomer with the triethylene glycol as side groups for the formation of micrometer-sized bundles of rigid superstructures, indicating the supramolecular aggregation would also be affected by the side group modification [30].

**Figure 3:**
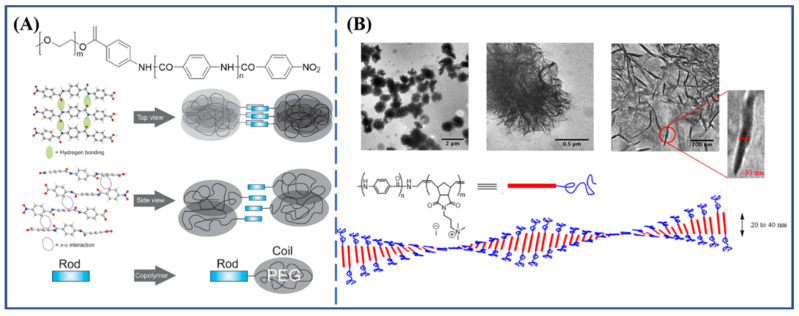
(**A**) Chemical structure and schematic self-assembly behavior of aramid-*b*-PEG [32]. Copyright 2010 American Chemical Society (Washington, DC, USA). (**B**) TEM pictures and schematic self-assembly behavior of amphiphilic aramid–ROMP block copolymer [34]. Copyright 2017 American Chemical Society.

The modification of end groups or introduction of a block segment into the main chain are also essential to achieve the self-assembly of aramids. Kilbinger and coworkers [31,32,33] reported the synthesis and aggregation behavior of the copolymer of an aramid oligomer with poly(ethylene glycol). The spherical or rodlike micelles’ number depended on poly(ethylene glycol) length in nonpolar solvents. The schematic self-assembly behavior of the block copolymer is shown in Figure 3A. Badoux et al. [34] prepared an amphiphilic aramid–ROMP block copolymer which self-assembled into large ribbonlike aggregates up to 50 nm in thickness and 300 nm in length (Figure 3B). Kataoka et al. [35] reported the gelation of monodisperse diblock copolymers of poly(*N*-octyl *m*-benzamide) with poly(*p*-benzamide) or poly(*m*-benzamide). The diblock copolymers could form spherical micelles or amorphous aggregates in many solvents. After the evaporation of solvents, the network-like patterns appeared because of the aggregation of the copolymer. Seyler et al. [36] reported aramid–peptide conjugates based on oligo(*p*-benzamide) as peptide amphiphiles to self-assemble into rod-like micelles in water.

**Figure 4:**
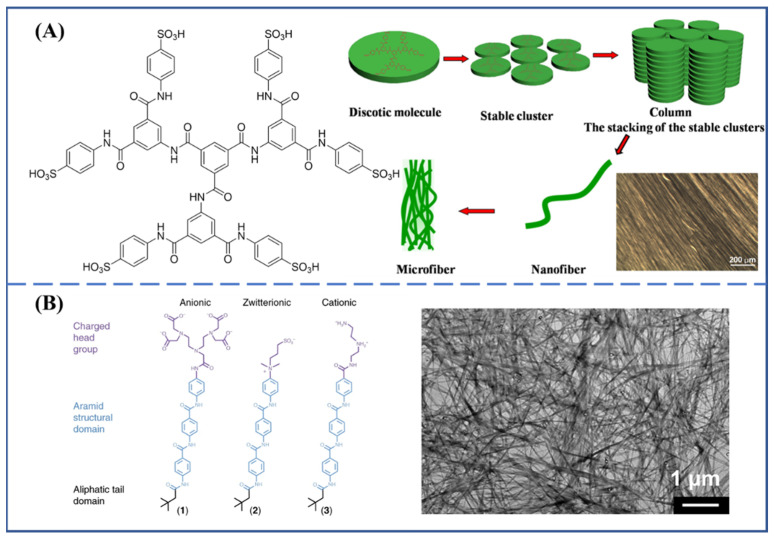
(**A**) The self-assembly behavior of discotic aromatic aramid molecule [37]. Copyright 2017 Elsevier B.V.; (**B**) The aramid molecule containing a charged head group and an aliphatic tail spontaneously self-assemble into nanoribbons in water [38]. Copyright 2021 Springer Nature (Berlin/Heidelberg, Germany).

In addition, small-molecule self-assembly based on aramid structure is also an established route for fabricating an ordered structure. Huang et al. [37] prepared a fully rigid, water-soluble, discotic-shaped aromatic aramid molecule. Through controlling the molecular states, such as solution, liquid crystals and solid state, the multiscale fibers that were nano- to micro-scale in both length and diameter could be self-assembled spontaneously (Figure 4A). Tempesta et al. [38] described the spontaneous self-assembly of aramid amphiphiles into nanoribbons upon the addition of water. The nanoribbons with a Young’s modulus of 1.7 GPa and tensile strength of 1.9 GPa could be extend to hierarchically ordered macroscopic materials outside of solvated environments (Figure 4B).

### 2.2. Polymerization-Induced Self-Assembly of PPTA

Rather than the pre-chemical modification and the post-self-assembly strategy proposed above involving generally complex synthetic routes for specially designed monomers, the direct self-assembly of PPTA into nano-objects during its polymerization, i.e., polymerization induced self-assembly, is another promising strategy that is especially attractive for practical applications.

**Figure 5:**
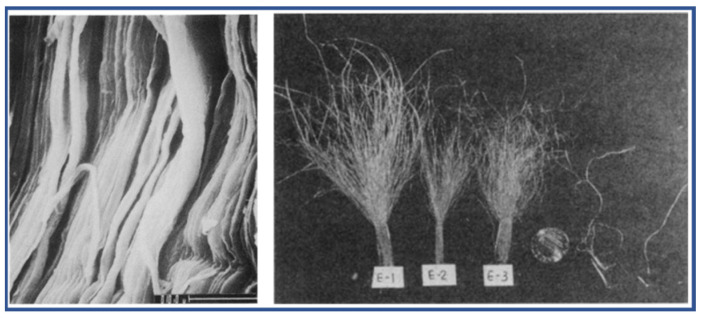
Growth-packed micron-sized PPTA fibers [40]. Copyright 1987 Springer Nature.

The early attempts were in the early 1980s, when Yoon et al. [39] reported the fabrication of aramid pulp via low-temperature-solution polycondensation of PPD with TPC in the NMP/CaCl_2_/pyridine solvent system. The rod-shaped PPTA molecular chains were growing and orienting along the shear force field direction in the reaction process until the PPTA gel formed in the reaction systems. The fibrillar polymer aggregation was precipitated after adding the precipitant. The products were pulverized, neutralized and washed with water to form an PPTA pulp with a certain aspect ratio and specific surface area. In 1987, Yoon obtained micron-sized PPTA fibers grown in a stationary gel phase containing active PPTA oligomer, heterocyclic tertiary amines, alkali metal cations and a polar solvent after the polymerization [40]. The process to fabricate the micron-sized PPTA fibers was described as the grown-packing method. The images of micron-sized PPTA fibers obtained by the grown-packing method are shown in Figure 5. The fibers were structurally similar to aramid pulp, and the fabrication process was facile without concentrated sulfuric acid spinning and fibrillation.

In 2016, Tuo’s group developed a novel self-assembly method to prepare the polymerization-induced aramid nanofiber (PANF) [41]. The researcher introduced methoxy polyethylene glycol (mPEG) into the copolymerization system as the dispersing agent for controlling the aggregation degree of PPTA molecular chains and stabilizing the formed aggregations. With the assistance of mPEG, PANF with the diameter range of 20–50 nm could be directly fabricated during the polymerization and dispersed uniformly in an aqueous system. Similarly to the chemically modified PPTA described above, the copolymerization of the flexible segments would result in a decrease in thermal resistance.

**Figure 6:**
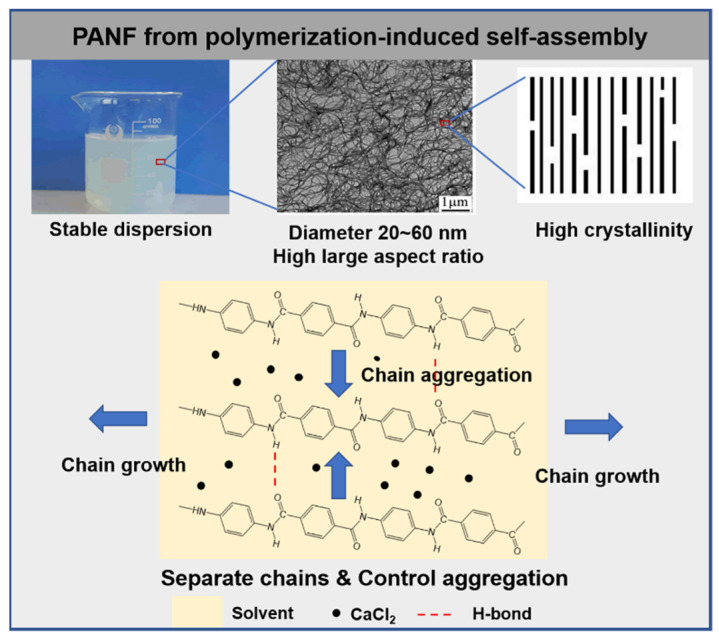
Fabrication of PANF via polymerization-induced self-assembly strategy.

For the purpose of eliminating the influence of chemically bonded mPEG, the nonreactive polyethylene glycol dimethyl ether (DME) was used to replace the reactive mPEG as the dispersing agent so that the added DME could be removed by the repeated washing and filtration [42]. The thermal stability of the obtained PANF was then significantly improved. In the recent report by the same group, an improved polymerization-induced self-assembly strategy was proposed which prepares PANF without using any dispersing agent or auxiliary outside the formulation list of PPTA polymerization [43]. As a heterogeneous means of polymerization, the growth of the molecular weight and the aggregation of PPTA molecular chain were carried out simultaneously until the gel finally formed. The presence of solvent and CaCl_2_ in the reaction system was utilized to adjust the hydrogen bonding association, and the hydrophilicity of these intrinsic solubilizers assisted the dispersing of the assembled nanofiber aggregation into aqueous mediums. Hence, the stable PANF could be obtained in an environmentally friendly aqueous dispersion form. The scheme of the PANF self-assembly process is described in Figure 6. The direct and time saving technology greatly simplifies the preparation process of aramid nanofibers and provides the possibility of industrialization. In China, Shandong Jufang New Material Co. Ltd. has built up a production line of PANF with an output of 300 tons/year.

**Figure 7:**
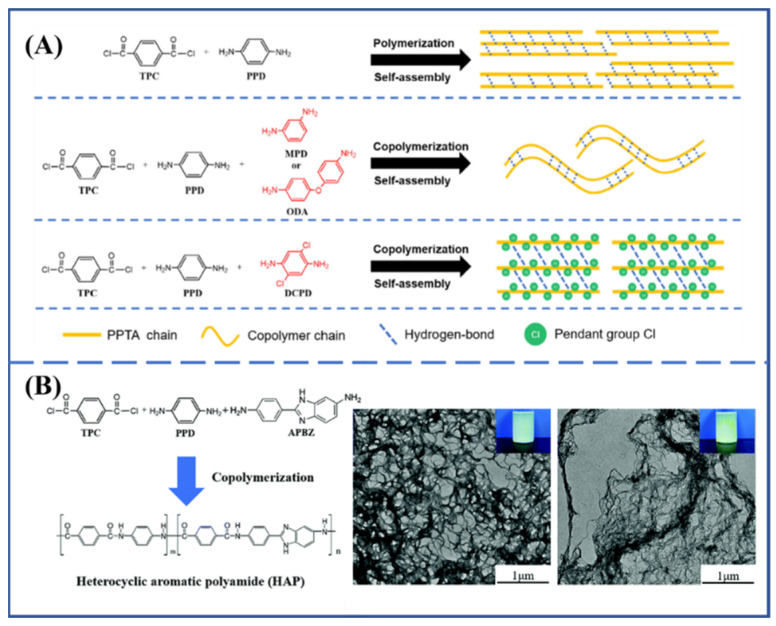
(**A**) Schematic self-assembly of copolymerized aramid nanofiber [44]. Copyright 2020 John Wiley and Sons (Hoboken, NJ, USA). (**B**) The synthesis of a heterocyclic aramid and the morphology of the heterocyclic aramid nanofiber [45].

In addition to the di-component copolymerization system of PPD and TPC, the polymerization-induced assembly strategy is also suitable for the fabrication of aramid nanofibers with the copolymerization of third co-monomers. In order to illustrate this, Shi et al. [44] separately introduced three kinds of third monomer, *m*-phenylenediamine, 4,4-diaminodiphenyl ether and 2,5-dichloro-1,4-phenylenediamine, into the polymerization-induced self-assembly process of PPTA, and nanofiber aqueous dispersions were all obtained with subtle differences in micromorphology (Figure 7A). The side groups of copolymerized aramids were of benefit for the decreasing of ANF diameter. Similarly, the third monomer, 2-(4-aminophenyl)-5-aminobenzimidazole, was also introduced to participate in the polymerization-induced self-assembly of PPTA, and the ribbon-like nanofiber network formed in the process of aggregation (Figure 7B). The introduction of heterocyclic monomer destroyed the chain’s regularity, but the thermal stability of the PPTA copolymer was further improved [45].

## 3. The Assembly of PPTA Nanofibers and the Applications

Owing to their intrinsic mechanical and thermal properties, and large surface area and aqueous dispersibility characteristics, PPTA nanofibers have drawn much attention as nano-building blocks for higher-order architectures, including films, hydrogels, aerogels, fibers and different kinds of composites. The enthusiasm started with the breakthrough work of Kotov’s group in 2011 [46]. Until now, different preparation approaches have been developed focusing on the “top-down” processing of commercial PPTA fibers (deprotonation [46], electrospinning techniques [47], mechanical disintegration [48], immersion rotary jet-spinning methods [49], etc.). The relevant achievements of the “top-down” PPTA nanofibers, especially those from the chemical cleavage methods, can be seen in recent reviews [50,51,52]. In this part, our discussion is mainly on the assembly of PANF toward the application of all-aramid materials.

### 3.1. Para-Aramid Paper

*Para*-aramid paper is an important high-performance polymeric material. Except for its high strength and excellent heat resistance, *para*-aramid paper has the highest insulation grade and high dielectric strength, and is widely used as an insulating material in electrical and electronic fields [53,54,55]. In addition, the aramid paper honeycomb can be prepared by the combination of *para*-aramid paper and adhesives through a specific process. Aramid paper honeycomb has the characteristics of low density and high specific strength, and is required as a weight-reducing material in important fields, such as aerospace, rail transportation, national defense and military industries [56,57,58].

**Figure 8:**
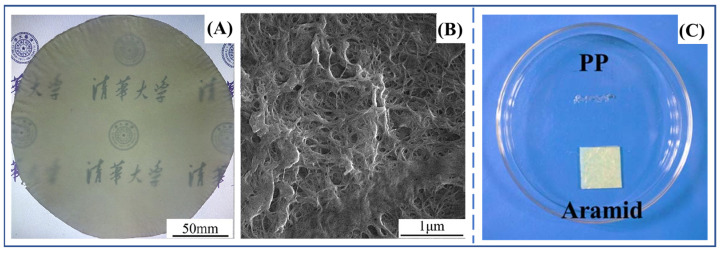
(**A**) Photograph of PANF paper. The Chinese words in subfigure is “Tsinghua University” [59]. (**B**) SEM image of PANF paper [59]. Copyright 2017 Elsevier B.V. (**C**) Photograph of PP separator and PANF separator after heated at 200 °C for 0.5 h [64]. Copyright 2016 John Wiley and Sons.

In the field of insulating materials, although traditional *para*-aramid paper exhibits excellent comprehensive properties, the tedious processing and the electrical breakdown strength need to be improved. The invention of PPTA nanofibers effectively addresses this problem. Tian et al. [59] prepared *para*-aramid paper from a PANF dispersion through a vacuum-assisted self-assembly method. The authors first converted a certain amount of PANF dispersion into thin PANF hydrogel film by filtration, and then the PANF hydrogel film was transformed into PANF paper under certain pressure and temperature conditions (Figure 8A). Microscopic scanning electron microscopy (SEM) analysis showed a relatively dense structure from the interface fusion of PANF driven by the drying process (Figure 8B), which endows PANF paper with excellent mechanical properties and electrical insulation properties. The tensile strength and heat resistance of PANF paper are both superior to those of commercial *meta*-aramid (Nomex) paper. The electrical breakdown strength of PANF paper was up to 86 kV/mm, much higher than that of Nomex paper and *para*-aramid paper prepared by other literature methods [53,54,55]. The preparation process of PANF paper is quite simple and has already been industrialized.

With the rapid development of lithium-ion batteries, recent studies have found that two-dimensional *para*-aramid paper (film) materials are also expected to be used in the new energy area [60,61,62,63]. Li et al. [64] prepared the PANF separator by vacuum-assisted self-assembly technology and evaluated it comprehensively according to the requirements of a lithium-ion battery separator. Compared with the commercial polypropylene (PP) separator, the PANF separator has a smaller contact angle and better wettability to the electrolyte. In addition to its superior mechanical properties, the heat resistance of PANF separator is also significantly superior to that of the PP separator (Figure 8C). These advantages indicate that the PANF separator’s potential for being applied in the field of lithium-ion batteries.

### 3.2. All-Aramid Structural Materials

In modern society, the demand for high-performance structural materials is increasing in the fields of construction, transportation, aerospace, military defense and industry [65,66,67,68]. The exploration of structural materials with the advantages of high specific strength, high toughness, high temperature resistance, wear resistance and corrosion resistance is the main direction for the development of a new generation of high-performance structural materials. PPTA is a typical representative of high-performance polymers. The *para*-aramid fiber prepared from PPTA has excellent properties, such as high strength, high modulus, high temperature resistance and corrosion resistance. However, the surface of *para*-aramid fiber is smooth and the interaction forces between the fibers are poor, so it is difficult to further process it into dense structural materials with a certain shape. If PPTA could be processed into structural materials, it would be expected to make full use of its performance advantages, and broaden the applications of aramid materials.

**Figure 9:**
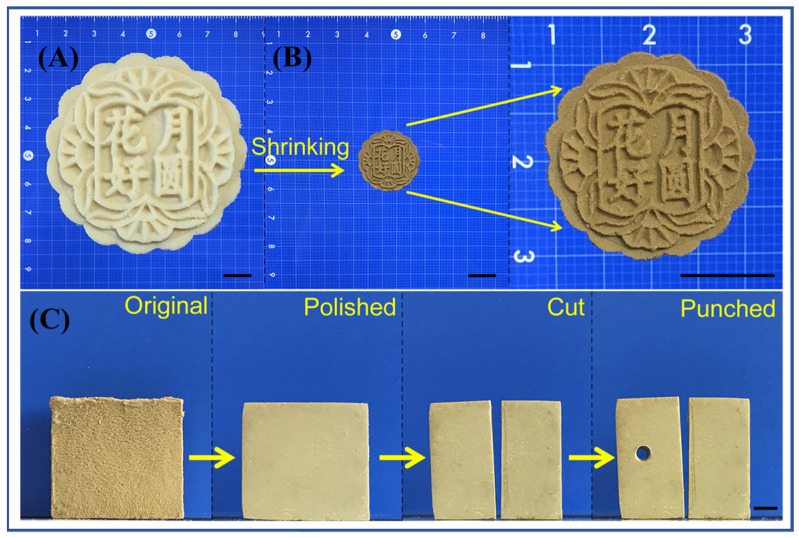
(**A**) PANF hydrogel in the shape of a mooncake; scale bar: 10 mm [43]. (**B**) PANF bulk in the shape of a mooncake; scale bar: 10 mm [43]. (**C**) Typical postprocessing approaches (polishing, cutting and punching) of PANF bulk; scale bar: 4 mm [43]. Copyright 2021 John Wiley and Sons.

Tuo et al. firstly concentrated the PANF dispersion to obtain the PANF hydrogel with a solid content of about 2%. The PANF hydrogel has good plasticity and can be molded into a certain shape with the help of a mold (Figure 9A). When the PANF hydrogel was dried at room temperature or by heating in an oven, an all-aramid bulk with a dense structure was obtained by evaporation-induced self-assembly finally (Figure 9B) [43]. The density of the all-aramid bulk can reach 1400 kg/m^3^, which is close to the density of *para*-aramid fibers. The tensile strength of the all-aramid bulk can reach more than 62 MPa, and the compressive strength can reach more than 90 MPa, which meet the mechanical strength levels of engineering plastics. Furthermore, the all-aramid bulk has excellent heat resistance and flame retardancy, better than most polymer materials. In addition to the direct preparation of special-shaped parts through mold forming, the all-aramid bulk can also be processed like wood by polishing, cutting, punching and other secondary processing methods (Figure 9C). Excellent properties and good processability make all-aramid bulk a promising high-performance structural material for harsh environmental conditions.

As mentioned above, aramid paper honeycomb, often used in aerospace and other fields, is also a kind of structural material with low density, high specific strength and excellent heat resistance. However, there are still many problems in traditional aramid paper honeycomb. For example, the preparation process of aramid paper honeycomb is complicated, and the introduction of adhesive reduces the overall toughness and heat resistance, and shortens the service life of the material. Additionally, the honeycomb has a single structure and cell walls of nonuniform thickness. These shortcomings limit the production and application of aramid paper honeycomb [56,57,58]. Except for the above shortcomings, the preparation of *para*-aramid paper is difficult, which further limits the preparation of *para*-aramid paper honeycomb. Additionally, the current commercialized aramid paper honeycomb is dominated by the *meta*-aramid paper honeycomb.

**Figure 10:**
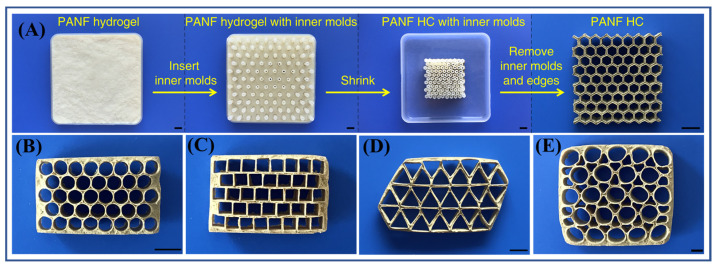
(**A**) The preparation process of PANF honeycomb (HC); scale bars: 10 mm [43]; (**B**–**E**) PANF HCs with different round, square, triangular and irregular cells, respectively; scale bars: 10 mm [43]. Copyright 2021 John Wiley and Sons.

Taking advantage of the feature that a PANF hydrogel can shrink uniformly and centripetally during the drying process, all-aramid honeycomb materials with various cell structures composed completely of PPTA can be prepared by the template method (Figure 10) [43]. As shown in Figure 10A, the preparation process of all-aramid honeycomb is simple: first, a certain concentration of PANF hydrogel is molded into a flat shape in a mold, and then the hexagonal columns are regularly inserted into the PANF hydrogel to form an array; after that, the PANF hydrogel with regular hexagonal columns is dried at room temperature; then, with the evaporation of water, the hydrogel shrinks uniformly, and the shape of hydrogel is controlled by the hexagonal columns, finally forming a regular, thin-walled honeycomb structure. For the all-aramid honeycomb, except for its simple preparation process, there are also some other advantages when compared with the current aramid paper honeycomb: (1) The forming of all-aramid honeycomb is based on the hydrogen bonding between molecules and does not involve the addition of adhesives. This integrated structure helps to improve the shear strength and lateral compressive strength of the honeycomb. (2) Since it is completely composed of PPTA molecules, the heat resistance of an all-aramid honeycomb is obviously better than that of the commercial aramid paper honeycomb. (3) The shape of forming mold is adjustable, so honeycombs with different cells and structural parameters can be prepared, including those with round, square, triangular and irregular cells (Figure 10B–E), and different cell sizes and densities. In addition, the all-aramid honeycomb has excellent mechanical properties, and its specific compression and specific shear strength can reach or even exceed the levels of the traditional aramid paper honeycomb.

### 3.3. Para-Aramid Aerogel

Aerogel is a kind of porous material with a network-like skeleton structure filled with a large amount of air. Due to their advantages of low density, high porosity, high specific surface area and low thermal conductivity, aerogels are widely used in many fields, such as thermal insulation, adsorption, filtration, catalysis and damping [69,70,71,72,73,74]. The earliest aerogel materials were inorganic silica aerogels, which have excellent heat resistance and low thermal conductivity, but are brittle and fragile. Organic polymer aerogels, such as polyurethane aerogels, have good toughness, but their heat resistance is poor and their application range is limited. Due to the high strength, high modulus and excellent heat resistance of *para*-aramid fibers, there have been many reports on the preparation of *para*-aramid aerogels in recent years [75,76,77,78]. However, the supercritical drying method was generally used in these reports, and the preparation process is long, complicated and difficult to be used for large-scale preparation.

**Figure 11:**
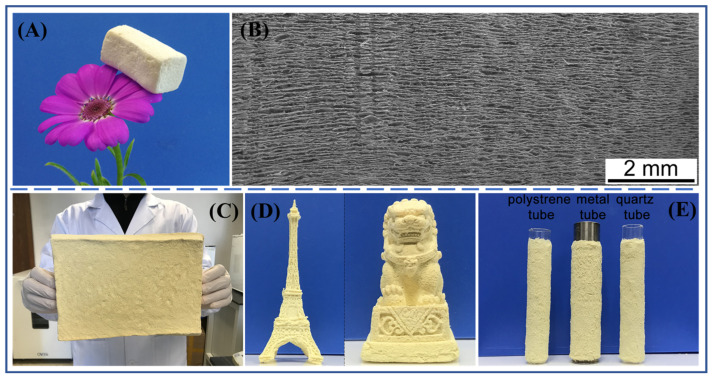
(**A**) Photograph of the laminated PANF aerogel [42]. (**B**) The inner structure of the laminated PANF aerogel [42]. Copyright 2019 American Chemical Society. (**C**) Large cuboid PANF aerogel (220 mm × 150 mm × 40 mm) [79]. (**D**) PANF aerogels with sophisticated shapes: left, Eiffel Tower; right, Chinese stone [79]. (**E**) Different materials coated with PANF aerogel. From left to right: polystyrene tube, metal tube and quartz tube [79]. Copyright 2021 American Chemical Society.

Using PANF dispersion as raw material, Xie et al. successfully prepared laminated PANF aerogel by combining vacuum-assisted self-assembly, ice-templated directional solidification and freeze-drying [42]. The density of the PANF aerogel was about 25 ± 2 kg/m^3^, and it could be placed stably on the petals (Figure 11A). The PANF aerogel has an anisotropic long-range ordered lamellar structure (Figure 11B), and the range of its ordered structure can reach more than 1 cm, which is difficult to be achieved with a conventional directional freezing processes. The formation of a laminated structure is attributed to the dual effects of vacuum-assisted self-assembly and ice-templated directional solidification on the orientational alignment of PANF. Vacuum-assisted self-assembly facilitates the formation of an inner layer-by-layer PANF network structure in the hydrogel, while the ice-templated directional solidification further promotes the orientation of the PANF network. Corresponding to its structure, the laminated PANF aerogel exhibits anisotropic properties, including mechanical properties and thermal conductivity. In the direction parallel to the laminae orientation, the compressive strength and specific strength of the aerogel are higher than those of most aerogels of the same density reported in the literature. In the direction perpendicular to the laminae orientation, the aerogel shows excellent resilience performance, and 95% of the original stress can still be maintained after 1000 compression rounds under 30% strain. In addition, the thermal conductivity of the direction parallel to the laminae orientation is slightly higher than that in the direction perpendicular to the laminae orientation, but both are less than 0.05 W/(m·K), showing its potential to be used as a thermal insulating material.

Although a laminated PANF aerogel has a regular structure and excellent comprehensive properties, the freeze-drying method used in the preparation process is not conducive to the large-scale production of PANF aerogel, and the directional solidification condition is a little complicated, which also hinders the batch preparation of PANF aerogels. Therefore, the authors further proposed a modified freezing-drying method, which can be used for the preparation of a PANF aerogel [79]. According to the method, a PANF hydrogel with a certain solid content was first frozen at −18 °C, and then the completely frozen PANF hydrogel was dried at room temperature to 150 °C to obtain the aerogel material finally. The PANF aerogel prepared by this method has obvious macroporous structure and is isotropic. Systematic studies have shown that PANF was phase-separated from water during the freezing process and formed a porous framework based on the strong hydrogen bonding between PANF. During the subsequent drying process, the framework is strong enough to support the pore structure and avoid its collapse, and finally, the aerogel structure is formed. This method is also essentially based on the mechanism of ice template-induced self-assembly. In the preparation process, both the freezing and drying processes can be carried out continuously, and the conditions are mild and make it easy to realize industrial-scale production.

In addition, PANF aerogels with different shapes and properties can also be prepared by this method. With the help of different molds, PANF aerogels that are large (Figure 11C), have complex shapes (Figure 11D) and are coated intimately around the curved surfaces of tubes (Figure 11E) can all be obtained. By adjusting the PANF hydrogel concentration and drying temperature, PANF aerogels with different densities and mechanical strengths can be prepared. PANF aerogel has a low thermal conductivity and can be used in thermal insulation, and its heat resistance is significantly better than that of commonly used commercial polyurethane foam. These advantages all increase the abundance of PANF aerogel products, which can further broaden their applications.

### 3.4. PANF/Polymer Composites

**Figure 12:**
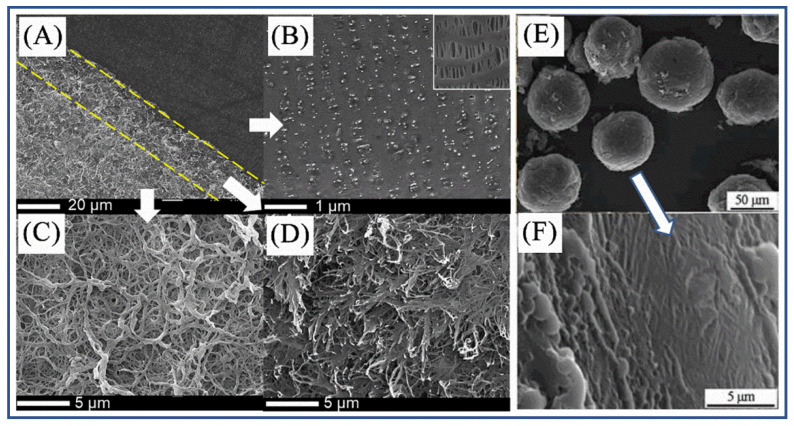
SEM images of PANF/polymer composites: (**A**) The vertical view of the entire PPTA@PPs separator. (**B**) PP substrate being peeled from the PPTA nanofiber coating layer. The inset is the original PP substrate. (**C**) Surface morphology of PPTA nanofiber coating layer. (**D**) Internal morphology of the PPTA nanofiber coating layer [80]. Copyright 2018 John Wiley and Sons. (**E**) SEM image of the PANF@PS composite microspheres. (**F**) The PANFs on the surfaces of the PANF@PS composite microspheres [81]. Copyright 2017 American Chemical Society.

In addition to preparing all-aramid products, PANF can also be used to prepare composite materials. He et al. [80] coated a PPTA solution on the surface of a PP separator, and then soaked the PPTA/PP composite film in a coagulation bath composed of certain proportions of NMP and water to precipitate PPTA, and finally obtained a PANF@PP composite separator (Figure 12A–D). The composite separator has two characteristics. First, it can realize the firm combination of PPTA and PP without adhesives. The principle is that the PPTA polymer solution can penetrate into the pores of the PP separator and physically bind with the porous substrate film when PPTA is precipitated under the action of the coagulation bath. Second, during the precipitation process, PPTA self-assembled into nanofiber networks, which not only maintained the structural integrity, but also did not affect the porosity of the separator.

Qiu et al. [81] synthesized PANF@polystyrene (PS) microspheres through the Pickering emulsion polymerization method and realized the self-assembly of PANF on the surfaces of PS microspheres. The authors first mixed styrene monomer with PANF dispersion and prepared an oil-in-water (styrene is the oil phase) Pickering emulsion by magnetic stirring. In the emulsion system, PANF was adsorbed at the interface as a solid emulsifier, providing a good isolation effect on the oil phase droplets to keep the droplets stable during the subsequent polymerization process. After polymerization, PANF-coated PS microspheres were obtained (Figure 12E,F).

Liu et al. [82] first prepared a laminated PANF aerogel framework by vacuum-assisted self-assembly, ice template directional solidification and freeze-drying method, and then the epoxy resin was combined with the aerogel framework by vacuum-assisted transfer method. Finally, the PANF aerogel/epoxy resin composite was successfully prepared after curing. The authors found that the tensile strength of PANF aerogel/epoxy resin composite was significantly higher than that of pure epoxy resin, which was attributed to the laminae orientation structure of PANF aerogel. Furthermore, the heat resistance of PANF aerogel/epoxy resin composites was better than that of pure epoxy resin.

## 4. Conclusions

In this review, the recent progress in the self-assembly and applications of PPTA-based polymers was briefly summarized. Being a well-known liquid crystal polymer, the potential of PPTA to be applied as an effective molecular unit for self-assembly has been increasingly addressed and attempted from the aspects of chemical structure modification and polymerization process optimization. The emerging concept of aramid nanofibers and their fast development suggest new possibilities for the fabrication of all PPTA macroscopic structures and the composites when the idea of hierarchical self-assembly has been added in. The results should be promising considering the performance advantages of *para*-aramids. However, the work is just beginning. There are still a lot of problems in the intrinsic mechanisms, process control and final applications. Challenges have been proposed for both the polymer scientists and the industrial engineers, but a bright future is worth expecting.
